# Comparison of Pre-Trained YOLO Models on Steel Surface Defects Detector Based on Transfer Learning with GPU-Based Embedded Devices

**DOI:** 10.3390/s22249926

**Published:** 2022-12-16

**Authors:** Hoan-Viet Nguyen, Jun-Hee Bae, Yong-Eun Lee, Han-Sung Lee, Ki-Ryong Kwon

**Affiliations:** 1 Intown Co., Ltd., No. 401, 21, Centum 6-ro, Haeundae-gu, Busan 08592, Republic of Korea; 2 Department of Artificial Intelligence Convergence, Pukyong National University, Busan 48513, Republic of Korea

**Keywords:** steel surface defect detection, YOLOv5, YOLOX, YOLOv7, Nvidia Jetson Devices

## Abstract

Steel is one of the most basic ingredients, which plays an important role in the machinery industry. However, the steel surface defects heavily affect its quality. The demand for surface defect detectors draws much attention from researchers all over the world. However, there are still some drawbacks, e.g., the dataset is limited accessible or small-scale public, and related works focus on developing models but do not deeply take into account real-time applications. In this paper, we investigate the feasibility of applying stage-of-the-art deep learning methods based on YOLO models as real-time steel surface defect detectors. Particularly, we compare the performance of YOLOv5, YOLOX, and YOLOv7 while training them with a small-scale open-source NEU-DET dataset on GPU RTX 2080. From the experiment results, YOLOX-s achieves the best accuracy of 89.6% mAP on the NEU-DET dataset. Then, we deploy the weights of trained YOLO models on Nvidia devices to evaluate their real-time performance. Our experiments devices consist of Nvidia Jetson Nano and Jetson Xavier AGX. We also apply some real-time optimization techniques (i.e., exporting to TensorRT, lowering the precision to FP16 or INT8 and reducing the input image size to 320 × 320) to reduce detection speed (fps), thus also reducing the mAP accuracy.

## 1. Introduction

In the workflow of producing industrial products, there are many factors, such as equipment, human, atmosphere, environment and processing technology, that causes defects on the surface of the product. Besides, surface defects lead to many side effects, for example, reducing the products’ quality and price and also increasing the chances of harmful and unstable effects on the following steps in the process workflow [1]. As a result, surface defects detector plays an important role in the production line. Nowadays, most inspections of defects are carried out by humans, whose efficiency varies from person to person. Also, the long duration working of eye observation often results in missing or wrong detection. The most significant drawback of manual detection is that the speed of detection is slower than that of the production line, which leads to operating the production line in low-speed mode, and reduces the efficiency of the whole workflow. Therefore, there are huge demands to find alternative solutions for manual inspection or replace human eyes with automated inspection systems. With the booming of computer vision and particularly deep learning, following object detection tasks, defects on the surface of a product can be considered as a specific object, which was applied in many industrial fields, e.g., wood, tiles, fabric, and steel. Defect detection has become a hot application that has drawn much attention from researchers and companies from all over the works. Among many industrial fields, steel production line plays an important role in heavy industry. Thus, many scholars have proposed methods for the detection and classification of steel surface defects with satisfying results. However, there are some fundamental problems of surface defects detection in general or particularly steel surface defect, namely real-time performance, small-scale dataset sample, small size target problem and unbalanced sample identification problem [2]. In addition, realscenarios in industrial production lines require real-time performance and an end-to-end system ranging from camera setup for image acquisition to devices for edge computing with deep learning models and screens to display detection and classification results for supervision. According to this problem, there is huge potential for improvement to meet demands from industrial production line perspectives. In this paper, we investigate the feasibility of using state-of-the-art one-state (SOTA) detector algorithms of the YOLO family to solve these problems mentioned above. Particularly, the contribution of this paper is as follows:

- Firstly, we compare the accuracy and speed of YOLOv5, YOLOX and YOLOv7 for real-time steel surface defects detectors. In detail, we conduct training experiments on these pre-trained models of the YOLO family with the transfer learning method on the NEU-DET dataset.
- Secondly, we deploy trained models on 3 devices to verify the feasibility of these models for real-time application, including the advanced high-computing PC GPU server with four RTX 2080, NVIDIA Jetson Xavier, and Jetson Nano to evaluate their real-time performance for steel surface defects detector.

The remainder of this essay is structured as follows. In Section 2, the literature review of steel surface defects detection based on deep learning is presented, which shows some limitations of previous works. In Section 3, we discuss the architecture of SOTA YOLO models, their specifics strong points and weaknesses. In Section 4, evaluation metrics are listed, followed by experiment setup. The training results and comparison will be introduced in Section 5. Then, we conclude the paper in Section 6.

## 2. Related Work 

In the scope of our study, we focus on real-time steel surfaces detector and study literature reviews according to this criterion as shown in Table 1. From literature reviews [2,3], there are two main approaches to object detection following deep learning methods according to detection accuracy and speed, including a two-stage and one-stage detector. The most popular method of a two-stage detector is Faster R-CNN (Faster Recurrent-Convolution Neural Network) [4], which utilizes a region proposal network (RPN) to generate regions of interest (ROIs) and then process classification and position regression on these ROIs. For example, ref. [5] proposed a lightweight version of Faster R-CNN by replacing feature extraction’s conventional convolution layer with depthwise separable convolution to boost the speed of detection by fours time and adding center loss to improve the model’s ability to classify different types of defects. This proposed network was trained on 4655 images of 6 types of defects, which attained an accuracy of 98.32% and an average inference time of 0.05 s per image (or 20 fps). In contrast, the representative methods of the one-stage detector are SSD [6] and YOLO [7], which utilizes a single network without RPN to detect ROIs to increase inference speed. Ref. [8] improved the YOLO network with 27 convolution layers, then evaluated on 1200 images of 6 types of steel strip surface defect, which achieved mean average precision (mAP) accuracy of 97.55% and detection speed of 83 FPS on GTX 1080Ti GPUs. Note that the dataset in [5,8] is not publicly available. At the same time, the following studies evaluated the same open-source steel surface defect, namely NEU-DET. Ref. [9] proposed CP-YOLOv3-dense, which is improved from the YOLOv3 baseline by implementing priority classification on the images, and replacing the two residual network modules with two dense network ones. Evaluation on NEU-DET, this method achieved mAP accuracy of 82.73 and the inference speed of 9.68 ms per image on GPU PG102 TitanX. Ref. [10] contributed a novel large-scale steel surface defect dataset, namely GC10-DET, and also proposed improved SSD with hard negative mining to encounter the problem of different scales and data imbalance of defects. The evaluation results on NEU-DET achieved an mAP accuracy of 72.4% and a detection speed of 27 ms on GPU RTX 2080Ti. Ref. [11] proposed IMN-YOLOv3 by replacing the conventional YOLOv3 backbone with MobileNetV2, introducing EFPN and FFM to detect multi-size defects, and adding IoU loss to encounter the difference between classification and bounding box-regression. The results on NEU-DET achieved an accuracy of 86.96% and a detection speed of 80.98 fps on the Tesla V100 GPU. Ref. [12] introduced a two-stage defects detector based on the proposed YOLOv5 and Inception-ResnetV2 models. This two-state framework achieved an accuracy of 83.3%, while improved YOLOv5 only got 78.1%. From the literature review, there are still potential improvements. For example, the YOLOv3 model is quite out of date, which was first published in 2018, and the real-time analysis was only carried out on advanced high-computing GPU servers, which did not take into account SoC devices or lightweight devices for application in real industrial scenarios. Also, there is a peer-reviewed comparison of YOLOv3 and modified YOLOv3, improved YOLOv5 (version 5.0), SSD, and Faster R-CNN. However, pre-trained YOLOv5 (version 6.0), YOLOv7 and YOLOX are yet to be compared for real-time application in steel surface defect detection. These YOLO models will be investigated and discussed in Section 3.

## 3. YOLO Models Architecture

**Figure 1:**
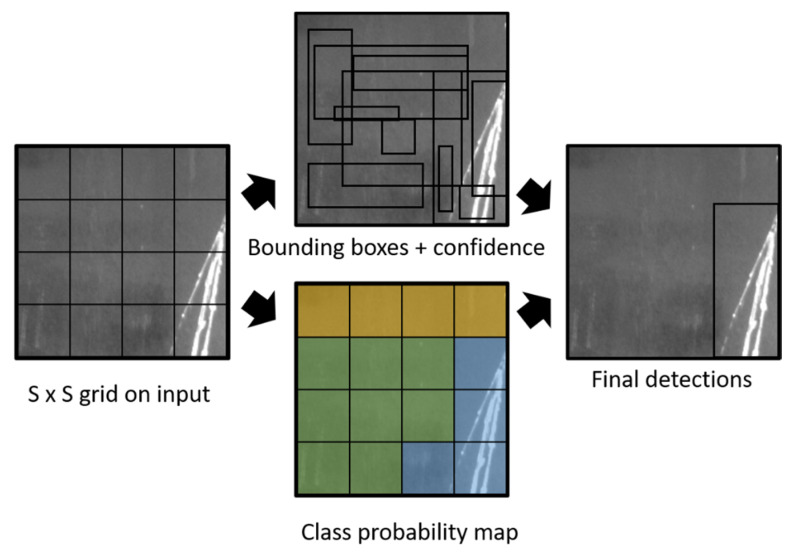
The overview of the YOLO algorithm.

Figure 1 presents the overview architecture of the YOLO algorithm. Following the different approach of conventional anchor-based models (e.g., Faster R-CNN [4] and SSD [6]), YOLO [7] performs the task of object detection as a regression task without ROIs detection. Instead, YOLO split the image into SxS grids to detect objects. If the object’s centroid appears in a grid, the grid is appointed to detect the object. In detail, YOLO predicts many bounding boxes with a different confidence score for each box. The confidence score indicates how likely the bounding box contains an object. Also, the YOLO predicts class probabilities per grid (regardless of the number of bounding boxes). Combining this information, YOLO makes final detections of corresponding bounding boxes. In summary, YOLOv1 is a fast single-stage object detector that is feasible for real-time application. However, there are several drawbacks of YOLOv1. For example, each grid can only have a class. If the small objects are very close to each other or two objects appear in the same grid, this architecture falls for small object detection. Also, the generalization performance of YOLO when training with a custom small-scale dataset encounters some problems. For example, the shapes of the bounding boxes are learned only through the training data, then YOLO is unable to accurately predict new and unique shapes of bounding boxes in the custom dataset.

In the next three versions of YOLO, from v2 to v4, each update encounters the limitation of the previous one by improving different parts of the model [13,14,15], mainly the backbone, the most important part of the YOLO structure. 

When YOLOv5 [16] was first released (YOLOv5 1.0), it can be considered as the PyTorch [17] implementation of YOLOv4 [15] instead of Darknet framework [18] and has a focus on exportability that can be deployed on various of environments. In 2022, the latest updated version of YOLOv5 is 6.1 (or YOLOv5 6.1), with many improvements; we will name them shortly as YOLOv5 in the following sections. For example, YOLOv5 nano is first released in version 6. The YOLOv5n model contained approximately 75% fewer parameters and also reduced size from 7.5 M to 1.9 M, which is ideal for lightweight devices (i.e., mobiles) and CPU solutions [16]. In YOLOv5 6 version, the model backbone architecture was significantly updated, e.g., the replacement of the Focus layer in version 1.0 with an equivalent Conv layer for improving exportability, replacement of the SPP layer with new SPPF layer, and reorder places of SPPF at the end of backbone [16]. Although YOLOv5 did not publish a peer-reviewed paper, through many updates, YOLOv5 has proved to be the most popular YOLO model in the YOLO family, which become the baseline of various improvements [12,19]. 

YOLOX [19] is another popular version of the YOLO family, which won the Streaming Perception Challenge CVPR 2021. YOLOX is a single-stage object detector that develops from the baseline of YOLOv3 with a DarkNet53 backbone and modified YOLOv5 backbone. Particularly, the head of the conventional YOLO baseline is replaced by a decoupled one in YOLOX. For each level of the feature map, a 1 × 1 Conv layer was used to reduce the feature to 256. Then, two parallel branches were added with two 3 × 3 Conv layers, which explains the name of the decoupled head. Each layer performs the task of classification and regression tasks respectively, which reduces the time convergence of training and the accuracy of the model. As opposed to conventional anchor-based YOLO algorithms, YOLOX removes the anchor boxes and follows an anchor-free mechanism, which broadens the deployment of YOLOX in SPC devices. 

YOLOv7 [20] is the latest SOTA object detector in the YOLO family, which will be released on June 2022 with a variety of structural reforms. The architecture is developed from the baseline of YOLOv4, Scaled YOLOv4, and YOLO-R. Also, the authors of these models were co-authors of YOLOv7. Particularly, YOLOv7 introduced E-ELAN (Extended Efficient Layer Aggregation Network) in the computational block of YOLOv7’s backbone and Model Scaling for Concatenation (MSC) based on different models, which helps to match these model requirements and make YOLOv7 more accessible in a variety of devices. As in [20], till June 2022, YOLOv7 proved to be the fastest and most accurate real-time object detector, which achieved AP accuracy of 56.8% and the inference speed from 5 to 160 FPS on various YOLOv7 models on the COCO dataset. 

Although there are other update variations in the YOLO family (e.g., YOLOv6 [21], PP-YOLO [22]), from deployment and exportability perspectives, we choose these three variations, YOLOv5 (6.1), YOLOX (3.0) and YOLOv7 (1.0) as pre-trained models. Firstly, these models support cache images before training, so it boosts the speed of training. Secondly, these models share the same native Pytorch training platform and support conversion from native training to another platform, such as ONNX or TensorRT, on their GitHub link, which means easy to customize the setup for install requirements and deploy applications on different devices, such as mobile and SoPC devices. In this work, we focus on the deployment of SoPC devices for verifying the feasibility of real-time steel surface detectors.

## 4. Experiments Setup

### 4.1. Training Environment

**Figure 2:**
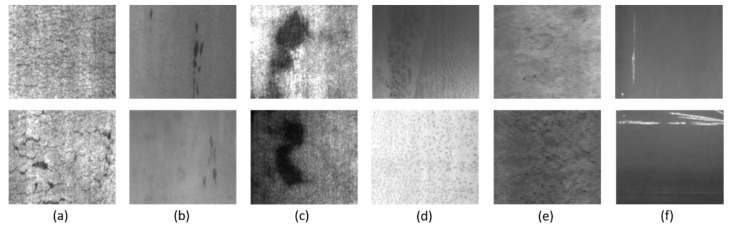
Examples of NEU-DET datasets include (**a**) Crazing, (**b**) Inclusion, (**c**) Patches, (**d**) Rolled-in Scale, (**e**) Pitted Surface and (**f**) Scratches.

We conduct experiments on the NEU-DET dataset [23] that consists of 6 defects categories on the surface of hot rolled steel strip, namely Rolled-in Scale (Rs), Patches (Pa), Crazing (Cr), Pitted Surface (Ps), Inclusion (In), and Scratches (Sc). There are 200 images for each type of defect, along with annotations files (or XML files), which contain information on bounding boxes and classes of defects in each image. Some parts of the dataset are shown in Figure 2, which indicates the complexity of the dataset and also the higher requirements for the detector for the classification task. 

For evaluation, we utilize the average precision (AP) of each defect and the mean average precision (mAP), precision (P) and recall (R) as the main accuracy evaluation metrics [24,25,26]. For example, the higher the mAP, the more accurate, and the smaller the mAP, the less accurate. In addition, to analyze real-time performance, we calculate the processing time per image as milliseconds in terms of frame-per-second (fps). The processing time per image includes pre-processing, inference and post-preprocessing time, which indicates the time of acquiring input image, time of detection, and time of non-maximum suppression (NMS), respectively.

For training, the YOLO models are trained with the NEU-DET dataset, which is divided into 80/20 percent for training and validation, respectively, as shown in Table 2. In order to evaluate those models’ accuracy performance fairly, we utilize the default settings of each model without changing any hyper-parameters and keep the same initialization parameters such as input image size, batchsize, and epochs. The epochs are initially set up to 100; the input image size is set as 640 × 640, and the batchsize is selected as 16. After each epoch, the evaluation metrics are calculated, including the Average Precise (AP) for each class, precision, recall, and the mean Average Precise (mAP). For future evaluation, the weights of each model are saved, and the best weight shall be used for deployment. For qualitative testing, the testing configurations are set up with an IoU threshold of 0.45, a confidence threshold of 0.25, and an image size of 640 × 640 as same as training. YOLO models are built, trained and tested by using 2 NVIDIA Graphics Processing Units (GPU) with 10 GB of memory and a 2.20 GHz Intel^®^ Xeon(R) Silver 4210 CPU. Other computer settings were described in Table 3. 

### 4.2. Deployment Devices Configuration

In order to test our model’s real-time performance, we deploy these YOLO-trained models into two Nvidia Jetson devices, including high-end GPU-based Nvidia Jetson Xavier AGX and low-end GPU-based Nvidia Jetson Nano. Deep learning and computer vision are supported by Nvidia Jetson devices, which are embedded AI computing systems with high performance and low power consumption. With the use of the NVidia JetPack SDK, which includes the TensorRT, OpenCV, CUDA Toolkit, cuDNN, and L4T with the LTS Linux Kernel, Jetson modules may be flashed [27]. The capabilities of Jetson AGX Xavier have significantly surpassed those of earlier Jetson modules, especially Jetson Nano. The detailed configuration of these two devices is listed in Table 4.

In addition, we conduct some real-time optimization techniques to reduce detection time. For example, we convert these YOLO models from native Pytorch training weights to TensorRT to deploy on NVIDIA devices. NVIDIA TensorRT is a high-performance inference optimizer and runtime that can be utilized to perform inference in lower precision (FP16 and INT8) on GPUs. Besides, while converting to TensorRT, we were able to select lower settings of precision (i.e., from FP32 to FP16 or INT8) and input image size (i.e., 640 × 640 to 320 × 320) to reduce inference time but lower the mAP accuracy.

**Figure 3:**
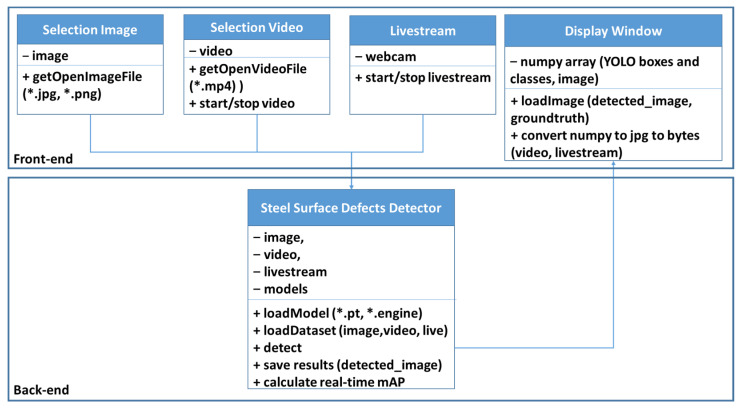
UML of our demo application.

Figure 3 shows the UML of the demo application, which is deployed on a server with GPU RTX 2080. This application is customized using YOLOv5 source code as a backend and programmed GUI front-end by PyQT5 and designer using Python language. The configurations of our developed application include Python 3.6 and PyQt5 version 5. We develop demo software using YOLOv5 source code because YOLOv5 has the 6th updated version over the last two years and is a well-supported platform. This demo application is also successfully deployed on Nvidia Jetson devices with some YOLOv5 source code customization based on our requirements. We set constraint types of image or video that which user can select, for example, "*.jpg" and "*.png” for image and “*.mp4”, while YOLOv5 can support other types of input data. YOLOv5 also supports loading livestream data by accessing to webcam’s camera. In backend detector, there are three important features, such as "loadModel”, “loadDataset” and “detect”, which are customized from YOLOv5 source code. YOLOv5 fully supports TensorRT “loadModel” and “detect” functions by their source code, it boost the time of deploying application. While the recently 3rd update version of YOLOX also support for TensorRT deployment, and YOLOv7 is recently released without fully support TensorRT deployment. 

**Figure 4:**
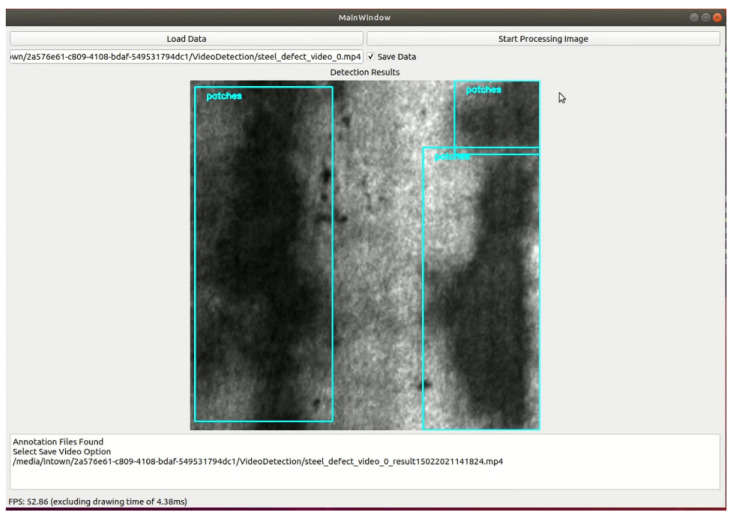
Example GUI of our demo application.

Figure 4 shows the GUI of our demo application with YOLOv5 as the backend. As can be seen from Figure 4, the user can click the button "Load Data" to select an image, the video then click "Start Processing Data" to detect using Steel Surface Defects Detector then display on the window screen. In the future work, we will develop our demo application by customizing YOLOX instead of YOLOv5 source code as backend and deploy our software on NVIDIA Jetson devices.

## 5. Experimental Results and Discussion

### 5.1. Training Models Results

In the training phase, these two smallest network size of each those three variations are built using pre-trained models weight of YOLOv5-n, YOLOv5-s, YOLOX-n, YOLOX-s, and YOLOv7-Tiny and YOLOv7 with transfer learning. The results of the AP and mAP@0.5 values in each model at an IoU threshold of 0.5 with a confidence threshold of 0.45 are shown in Table 5. It is noted that, several evaluation metrics (i.e., P and R in case of YOLOX) are not calculated by their released source code, which are stated as “-/-” in Table 5. From the pre-trained model weights property, including layers, parameters, GLOPs and size, it can be clearly seen that the most lightweight model are YOLOX-n with 0.9 M parameters only, while the YOLOv7 is the most computational complexity model with 37.2 M parameters. This pre-trained model weights information of these two models also reflex the reverted trend of inference time (fps) RTX 2080 as 240 fps and 119 fps respectively. Note that, the inference time in fps are calculated by using the best saving weights of these model with native Pytorch training platform on their public github link without any conversion.

As in [20], YOLOv7 has the best mAP results on COCO dataset with 80 classes. However, when these pre-trained models are trained on the custom dataset, particularly in this work steel surface defects NEU-DET dataset of 6 classes, YOLOv7 falls behind both YOLOv5 and YOLOX. In NEU-DET dataset, the most difficult classes for classification are crazing (Cr) and rolled-in-scale (Ro) ones. As can be seen in Table 5, the AP@0.5 of these two Cr and Ro classes are very low, e.g., YOLOv5-n only get AP of Cr and Ro as 40.1% and 64%. These two low AP of two class Cr and Ro leads to the mAP@0.5 of all 6 defects reduces significantly. In contrast, patches and scratches are much more easier to classify. For example, YOLOv5-n achieve AP for these two Pa and Sc classes as 90.5% and 91.4% respectively. YOLOX-s achieves the best mAP@0.5 on custom dataset NEU-DET with accuracy of 89.6%, which achieved by successful classification of Cr, Ro, Pa and Sc as 72.79%, 87.65%, 99.25% and 98.3% respectively. YOLOX family (YOLOX-n and YOLOX-s) proves to be the best NEU-DET steel surface defects classifier with mAP@0.5 of 82.4 and 89.6 respectively. In case of YOLOv5 family, YOLOv5s leads the accuracy of 1.3% compared to YOLOv5-n but YOLOv5-s parameters are four times higher than YOLOv5-n. By compensation between 1.3 % accuracy and 20 fps of inference times versus YOLOv5-n, YOLOv5-n is quite promising on deploying on devices and proves to be a good model on the release 6th update of YOLOv5. In case of YOLOv7 family, YOLOv7-Tiny fells behind with only 1% less than YOLOv7 at 72.4 % accuracy but much more faster than YOLOv7 with nearly 50 fps. 

**Figure 5:**
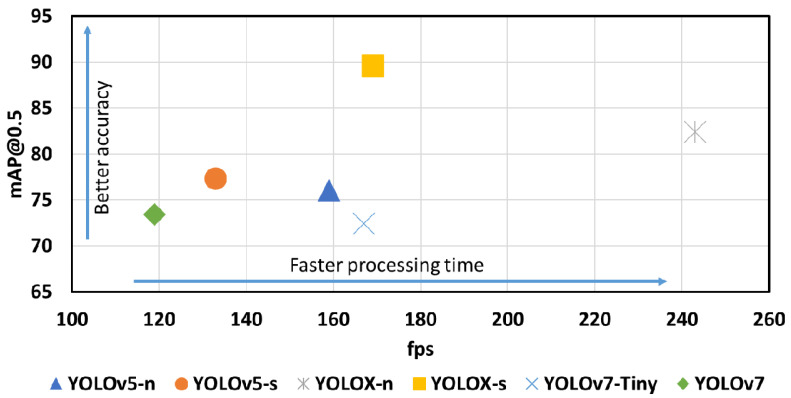
Comparison training results of six YOLO models in term of accuracy mAP@0.5 percentage and processing time fps on RTX 2080.

Figure 5 shows the qualitative comparison training results of six pre-trained YOLO models on NEU-DET dataset in term of accuracy mAP@0.5 and processing tim fps on GPU RTX 2080. As can be seen in Figure 5, the fastest YOLO model is YOLOX-n while the slowest one is YOLOv7. The model achieves the best accuracy of mAP@0.5 is YOLOX-s while YOLOv7-Tiny get the lowest accuracy. 

**Figure 6:**
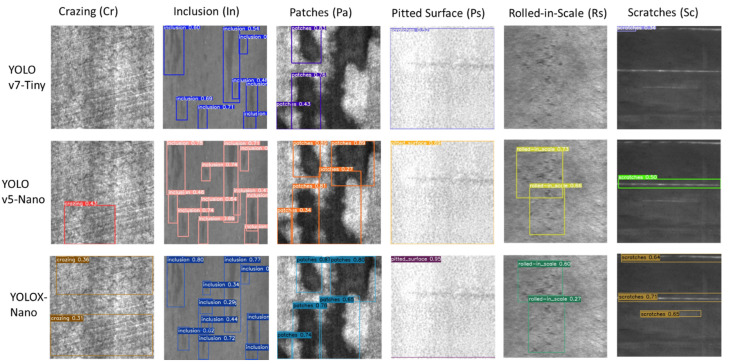
Qualitative Results of three pre-trained YOLO models, including YOLOv7-Tiny, YOLOv5-Nano, and YOLOX-Nano.

Figure 6 shows the qualitative results of three pre-trained YOLO models, namely YOLOv7-Tiny, YOLOv5-Nano and YOLOX-Nano. As can be seen from Figure 6, in some test images, YOLOv7-Tiny cannot detect the Crazing and Rolled-in-Scale defects, wrongly classifies Pitted Surface defect as Scratches, and miss detection some Inclusion and Scratches defects. In comparison between YOLOv5-Nano and YOLOX-Nano, YOLOX-Nano proves to be better detection of Crazing and Scratches than YOLOv5-Nano, which helps YOLOX-Nano have better accuracy of mAP@0.5 than that of YOLOv5-Nano.

### 5.2. Deployment on Devices Results

As mentioned before, while deploying on two NVIDIA Jetson devices, namely Xavier AGX and Nano, we convert these models best weights from native Pytorch training platform to TensorRT. Table 6 shows the comparison of these YOLO models TensorRT weights in terms of model size storage and inference time on two NVIDIA devices. As can be shown in Table 6, the smallest model size is YOLOX-n at 4.7 MB, that follows by YOLOv7-Tiny and YOLOv5-n at 15 and 19.6 MB respectively. The two version nano and small of YOLOX models, namely YOLOX-n and YOLOX-s are the smallest model size compared to their corresponding versions of YOLOv5. While the largest model size belongs to YOLOv7 at 135 MB. 

Note that the inference time is calculated in millisecond from these models default Github source code, which we convert to fps for easier real-time evaluation. NVIDIA Jetson Xavier AGX is a very powerful device, which YOLOX and YOLOv5 family can achieve real-time performance with highest fps of 48 and lowest fps of 23. In the case of YOLOv7, the authors report that YOLOv7 is not suitable for mobile device deployments. It can be seen from Table 6 YOLOv7 requires very high computation resources. Also, YOLOv7 proves to be not feasible for real-time detectors when deploying on NVIDIA Jetson devices, Xavier and Nano, with very low average fps of 17 and 3 respectively. At the same time, the real-time performance of YOLOv7-Tiny on Jetson Xavier is very promising, with an fps of 40, which is similar to YOLOX-n. 

In this work, we also take into account low-cost devices like Jetson Nano for real-time performance evaluation. The nano weights size of YOLOv5 and YOLOX and the tiny weight of YOLOv7 achieves 18, 13, and 16 fps, respectively. For low-cost devices like Nano, these fps are feasible for nearly real-time object detectors. As for small weight size, both YOLOv5 and YOLOX achieve around 9 fps, while YOLOv7 falls behind at 3 fps, which is not suitable for the real-time detector. 

**Figure 7:**
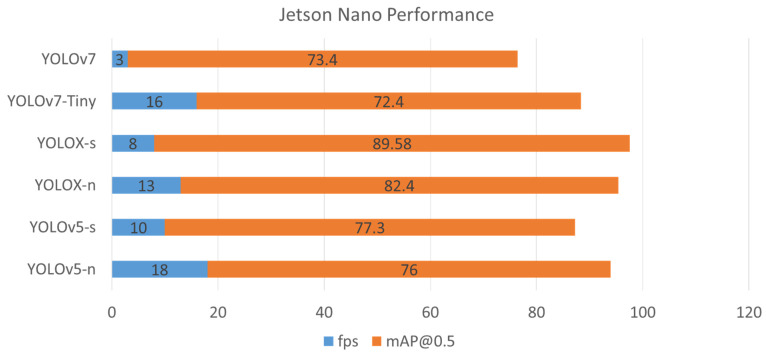
Jetson Nano performance of six pre-trained YOLO TensorRT models.

Figure 7 presents the combination performance of accuracy mAP@0.5 and inference time fps on the Jetson Nano device. To compensate for these two factors, YOLOX-s proves to be the best detector with the highest points of 97.6, then YOLOX-n and YOLOv5-n fall behind shortly with 95 and 94, respectively.

In real industrial scenarios, considering the distance between the camera and the production line from 50–100 cm and the typical maximum speed of the running line at about 30 m per second (m/s), the inference speed of the device should be at least from 30 to 60 fps [8]. From the results of the experiments, it can be seen that the high computing device (i.e., Jetson Xavier) can achieve real-time performance (i.e., more than 30 fps) with several YOLO-TensorRT models (i.e., YOLOX-s, YOLOv5-n, YOLOv7-Tiny). While the low computing device (i.e., Jetson Nano) fails to fulfill the real-time performance, the detection speed at 18 fps of YOLOv5-n and 16 fps of YOLOv7-Tiny are quite impressive compared with the affordable price of Jetson Nano. It is noted that, in this work, we only export the YOLO weight from native Pytorch to TensorRT while keeping the precision at FP32 and input image size of 640 × 640 similar as training configurations, to keep the mAP accuracy. In the case of reducing the precision from FP32 to FP16 or INT8 and input image size from 640 × 640 to 320 × 320, we can boost the detection speed but lower the mAP accuracy. In summary, the cost of hardware is very important for factory applications. Besides, not only the hardware but also the model is a crucial factor as well. As the model complexity increase, higher computing power is necessary to maintain or reduce inference time. However, there is a constraint of upgrading hardware to increase computing power under the cost limitations (funding). Thus it is necessary to choose the model and hardware according to computing power, cost and inference time depending on the defined problem or analysis purpose with data (i.e., the steel surface defect detection scenarios in this work).

## 6. Conclusions

In this work, we verify the performance of stage-of-the-art YOLO family models for the task of real-time steel surface defect detection and classification in terms of accuracy and real-time analysis. The motivation of this work is also to testify to the feasibility of deploying YOLO models on devices for smart-factory applications. We conduct a comprehensive experiment from YOLO models comparison and deployment application for real-time steel surface defect detector, which boosts the time of further research and bridges the gap between research and factory. Apart from these fundamental features, we customize the application with other options, such as saving detected results in the format of image or video according to input data and calculating real-time mAP accuracy, which can be integrated into the smart-factory application.

## Figures and Tables

**Table 1:** Related work of steel surface defect detection algorithms.

Reference	Dataset	Algorithms	Results
Ref. [12] 2022	Enriched-NEU-DET 2224 images Train/Test/Validation Set: 6/2/2 Image Size: 576 × 576	Improved YOLOv5	78.1%
Ref. [11] 2022	Conventional NEU-DET Train: Testing: Resolution:416 × 416	IMN-YOLOv3-Pytorch	86.96% 80.959 fps (GPU Tesla V100)
Ref. [10] 2020	Conventional NEU-DET	Improved SSD with negative hard mining	72.4% 27 ms (GPU RTX 2080Ti)
Ref. [9] 2020	Relabeled Crazing defects Of Conventional NEU-DET	CP-YOLOv3-DarkNet	82.73% 9.68 ms (GPU GP102 TITAN X)
Ref. [8] 2018	Private Dataset	Improved YOLO	97.55% 83 fps (GPU RTX 1080Ti)
Ref [5] 2018	Private Dataset	Slighter Faster R-CNN	98.32% 20 fps

**Table 2:** Training/Testing Configuration.

Dataset Number	Training/Testing Set	Defect Name	Defects
1800 images	Training set (1440 images)	Crazing	
		Inclusion	
		Patches	
		Pitted Surface	
		Rolled-in Scale	
		Scratches	
	Testing set (360 images) 845 labels	Crazing	137
		Inclusion	190
		Patches	189
		Pitted Surface	79
		Rolled-in Scale	137
		Scratches	113

**Table 3:** Training/Testing setup environment on GPU server.

Device	Configuration
Operating System	Ubuntu 20.04
Processor	Intel^®^ Xeon(R) Silver 4210 CPU @ 2.20 GHz × 40
GPU	RTX 2080 10 G × 2
GPU accelerator	CUDA 11.2, Cudnn 8.1
Framework	PyTorch 1.9.1
Complier IDE	Pycharm
Scripting language	Python 3.6

**Table 4:** Deployment devises configuration.

Devices/ Configurations	NVIDIA Jetson Xavier AGX	NVIDIA Jetson Nano
AI Performance	5.5–11 TFLOPS (FP16) 20–32 TOPS (INT8)	0.5 TFLOPS (FP16)
CPU	8-core NVIDIA Carmel Arm^®^v8.2 64-bit CPU 8 MB L2 + 4 MB L3	Quad-Core Arm Cortex-A57 MPCore
GPU	512-core NVIDIA Volta™ GPU with 64 Tensor Cores	128-core NVIDIA Maxwel GPU
DL accelerator	2x NVDLA v1	N/A
Memory	64 GB 256-bit LPDDR4x 136.5 GB/s	4 GB 64-bit LPDDR4 25.6 GB/s
Price	$99	$699

**Table 5:** Performance of six models on NEU-DET validation set on RTX 2080.

Pytorch Models Weights	Pre-Trained Model Parameters	AP@0.5						mAP@0.5	P	R	fps
		Cr	In	Pa	Pi	Ri	Sc				
YOLOv5-n	280 layer, 3M paras, 4.3 GFLOPs 6.7 MB	40.1	87.3	90.4	82.7	64.0	91.4	76.0	77.3	70.7	159
YOLOv5-s	280 layers, 12.3M paras, 16.2 GLOPs 25.2 MB	46.1	82.2	91.1	87.8	64.9	91.8	77.3	76.6	73.0	133
YOLOX-n	-- 0.91M paras, 1.08 GLOPs 7.6 MB	60.6	86.8	90.1	82.2	76.1	98.3	82.4	-/-	-/-	243
YOLOX-s	-- 9M paras, 26.8 GFLOPs 71.8 MB	72.8	90.2	99.3	89.3	87.7	98.3	89.6	-/-	-/-	169
YOLOv7-Tiny	263 layers, 6M paras, 13.2 GLOPs 12.3 MB	37.0	82.8	87.8	82.3	55.5	89.0	72.4	65.8	72.8	167
YOLOv7	415 layers, 37.2M paras, 104.8 GLOPs 74.9 MB	36.8	85.6	88.1	80.7	58.7	90.4	73.4	68.3	73.7	119

**Table 6:** Comparison of different YOLO-TensorRT models in term of size storage and inference time on NVIDIA Jetson devices.

TensorRT Models Weight	Model Size (MB)	NVIDIA Jetson Devices	Inference Time (ms)	FPS
YOLOv5-n	19.6	Xavier AGX	20	48
		Nano	54.7	**18**
YOLOv5-s	66.2	Xavier AGX	44	23
		Nano	99.9	**10**
YOLOX-n	4.7	Xavier AGX	24.87	40
		Nano	78.13	**13**
YOLOX-s	21.5	Xavier AGX	31.64	32
		Nano	128.87	**8**
YOLOv7-Tiny	15	Xavier AGX	25.1	40
		Nano	63	16
YOLOv7	135	Xavier AGX	58.6	17
		Nano	319	3

## Data Availability

The NEU-DET is available online at http://faculty.neu.edu.cn/songkechen/zh_CN/zdylm/263270/list/index.html (accessed on 12 December 2022).
